# Curcumin versus triamcinolone acetonide gel in treating minor recurrent aphthous stomatitis: A randomized trial

**DOI:** 10.6026/973206300215050

**Published:** 2025-12-15

**Authors:** Harsh Prakash Srivastav, Ankita Jain, Pradeep Tangade, Vikas Singh, Ishmita Jain, Gaurav Kumar

**Affiliations:** 1 Department of Public Health Dentistry, Teerthanker Mahaveer Dental College and Research Centre, Moradabad, Uttar Pradesh, India

**Keywords:** Curcumin, healing, pain reduction, recurrent aphthous stomatitis, triamcinolone acetonide

## Abstract

The effect of Curcumin gel and Triamcinolone Acetonide gel to cure tiny recurrent aphthous stomatitis (RAS) is of interest. Hence, 60 patients in groups divided evenly between 18 and 40 years old are observed for seven days the research showed that both treatments reduced pain, ulcer size and healing time of wounds significantly. But Curcumin gel had a slight edge with fewer side effects. Results of the tests found faster healing and better patient satisfaction in the Curcumin group. Thus, we show the demonstration that Curcumin gel is a safer and more effective alternative to Triamcinolone Acetonide gel for treating minor recurrent aphthous stomatitis.

## Background:

Recurrent aphthous stomatitis, or RAS, is a general condition involving painful and recurring ulcers in the non-keratinized oral mucosa. Despite the fact that it is benign, RAS still requires a great deal out of people both physically and emotionally [1]. The ailment is widespread, with approximately 5 to 25 percent of the globe's population suffering from minor aphthae of some form [2]. The condition peaks in young adults and is thus of interest to doctors. Recent studies in India have found that minor aphthae are the most common form, thereby increasing the clinical burden and the need for effective, convenient treatment suitable to different levels of healthcare [3]. Minor RAS usually appears as one or more shallow, well-defined, round or oval ulcers, surrounded by an erythematous halo and with yellowish-gray pseudomembranes [4]. These ulcers generally heal in 10-14 days without scarring, but their frequency varies, occurring every week, a few months, or yearly again and again [5]. Major aphthae, however, are more persistent and heal with scarring; herpetiform aphthae present as myriads of tiny vesicular lesions which may become confluent. Each type of aphthae has characteristic healing times, morbidity levels and therapeutic needs [6]. Pain due to RAS can greatly affect eating, speaking and oral maintenance. When many ulcers appear at the same time or start to recur at regular intervals, they can have a substantial impact on the quality of life and nutritional intake of patients with this disease [7]. The exact cause of RAS is not well known, but it is generally regarded as an array of multi-factorial disease, so there are many contributing factors. These include mucosal damage, stress, poor nutrition, genetic susceptibility and disordered immune responses. Research has shown that several inflammatory mediators such as interleukin 6 (IL-6), tumor necrosis factor-α (TNF- α) and interleukin 2 (IL-2) are important in the occurrence of RAS [8]. These proinflammatory cytokines are implicated in the T cell-driven epithelial damage that is characteristic of the disease. Updated reviews underline the importance of these cytokine networks, which may provide targets for anti-inflammatory treatments and immunomodulation [9]. The standard treatment for RAS is topical corticosteroids, the first-line choice for alleviating symptoms. They help to relieve pain, accelerate healing and reduce the severity of ulcers. In significant or refractory cases, systemic steroids may be used [10]. Other measures, such as chlorhexidine and benzyldiamine mouthwashes, anesthetics, tetracycline steroid combinations, can make life more comfortable and reduce the chance of secondary infection, but cannot be relied upon to prevent recurrence [11]. Treatment will be determined based on the severity and frequency of attacks, taking into account the patient's tolerance and any comorbid conditions. However, long-term steroid use can have side effects, including oral candidiasis which requires antifungal treatment [12].

Triamcinolone acetonide is available in 0.1% concentration topical preparations, often in an adhesive base, such as Orabase. Triamcinolone helps reduce the pain and swelling associated with the sores. It is a well-established corticosteroid used for treating minor RAS [13]. Because it has both anti-inflammatory potential (with good substrate contact time) on top of that mucosal adhesion ability (prolonging residence-time for its constituents like corticosteroid calories), Triamcinolone provides an effective treatment option in RAS [14]. However, corticosteroids are not without their dangers. Both mucosal atrophy and oral fungal infections have been reported with long-term use. This has led to interest in finding alternative means for treating recurrent cases of RAS, ideally without resorting to corticosteroids in any form [15]. Curcumin, an active polyphenol extracted from Curcuma longa, is being explored as a potential alternative to steroids for treating RAS. Curcumin is known for its anti-inflammatory, antioxidant, immunomodulatory, antimicrobial and wound-healing properties [16]. It is likely to have an inhibiting role in the underlying inflammation, contributing to RAS. In particular, it targets such inflammatory pathways and cytokines as TNF-α and IL-6 which are central mediators in mucosal pain and injury in RAS. A curcumin 0.05% gel or the orabase form of the agent can be applied topically [17]. This ensures targeted effect while minimizing systemic exposure. Curcumin is thus an attractive option when managing the recurrent and self-limiting nature of this condition, providing a non-steroidal approach to symptomatic relief [18]. Therefore, it is of interest to determine the comparative effectiveness of Curcumin and Triamcinolone Acetonide gels in the treatment of minor RAS, evaluating their impact on pain reduction, ulcer size, healing time, adverse effects and patient satisfaction.

## Methodology:

This study was designed as a comparative clinical trial to assess and compare the effectiveness of Curcumin gel versus Triamcinolone Acetonide gel in the treatment of minor RAS. A total of 60 patients, aged between 18 and 40 years, who had minor RAS measuring between 1 to 5 mm, were selected for inclusion in the study. These patients were randomly assigned to two groups: the experimental group (Curcumin Group) and the control group (Triamcinolone Acetonide Group). In the experimental group, patients received the application of Curcumin gel on the ulcer, while those in the control group were treated with Triamcinolone Acetonide gel. The patients were then examined on Day 0, Day 3, Day 5 and Day 7 to monitor the regression of ulcer size and number and to assess any discomfort caused by the gel treatment. A randomized clinical trial design was used for this study. The source of data included the randomization of subjects into the experimental and control groups. The study was conducted among 60 patients from the Department of Public Health Dentistry. The participants were all aged between 18 and 40 years and diagnosed with minor RAS. The study was carried out at Teerthankar Mahaveer Dental College and Research Centre in Moradabad. Before commencing the study, a priori power analysis was conducted using G*Power software to determine the optimal sample size for comparing the means of two independent groups using a two-tailed t-test. The study assumed a large effect size (Cohen's d = 0.85), a significance level (α) of 0.30 and a desired statistical power of 0.80. Based on these parameters, the required sample size was calculated to be 60 participants, with 30 participants in each of the two groups. Ethical clearance for the study was obtained from the Ethical Committee of Teerthanker Mahaveer Dental College and Research Center, Teerthanker Mahaveer University, Moradabad, ensuring that the study adhered to all necessary ethical guidelines. The study was conducted over a period of 30 days, during which all participants provided written informed consent to participate, as outlined in Annexure I. The inclusion criteria for the study required participants to be aged between 17 and 40 years and have 1 to 5 minor aphthous ulcers with duration of less than 48 hours. Patients were excluded from the study if they had any systemic illness, major aphthous ulcers larger than 6 mm, ulcers located in inaccessible areas, traumatic ulcers, denture stomatitis, or if they were pregnant. Data collected during the study were presented as mean ± standard deviation (SD) for quantitative variables that followed a normal distribution. The significance of differences between groups was considered statistically significant at P < 0.05. Descriptive statistics were used to summarize the background and demographic data of the participants. To evaluate the efficacy of the two treatment modalities, the Mann-Whitney U test was used for statistical analysis. All data analysis was carried out using the Statistical Package for the Social Sciences (SPSS) software. Depending on whether the data followed a normal distribution, both descriptive and inferential statistical methods were employed to evaluate the study's outcomes.

## Results:

The results of this study are presented in the following tables, comparing the efficacy of Curcumin gel and Triamcinolone Acetonide gel in treating minor RAS. Data analysis was conducted based on patient assessments on Day 0, Day 3, Day 5 and Day 7, focusing on the reduction in ulcer size, pain relief and any discomfort caused by the treatment. The statistical analysis was performed using the Mann- Whitney U test and the significance level was set at P < 0.05. Table 1 (see PDF) presents the demographic characteristics of the 60 participants, which were evenly distributed between the two treatment groups (Curcumin and Triamcinolone Acetonide). The mean age, gender distribution and other relevant characteristics were comparable between the two groups, ensuring that the groups were similar at baseline. Table 2 (see PDF) shows the reduction in pain intensity as reported by patients in both groups. Pain intensity was measured using a Visual Analog Scale (VAS) and there was a significant reduction in pain in both treatment groups by Day 7. However, the Curcumin Group showed a slightly greater reduction in pain compared to the Triamcinolone Acetonide Group. The reduction in pain intensity was significant in both groups by Day 7 (P < 0.05), but the Curcumin Group showed a greater improvement in pain relief compared to the Triamcinolone Acetonide Group. Table 3 (see PDF) compares the reduction in ulcer size between the two treatment groups. The ulcer size was measured on Days 0, 3, 5 and 7. Both treatments led to a reduction in ulcer size, but the Curcumin Group exhibited a slightly more rapid reduction. Both groups demonstrated a significant reduction in ulcer size by Day 7; however, the Curcumin Group exhibited a greater reduction in size (P < 0.05). Table 4 (see PDF) illustrates the average time required for complete healing of the ulcers in both treatment groups. The Curcumin Group showed a faster healing time compared to the Triamcinolone Acetonide Group. The Curcumin Group showed faster healing, with a statistically significant difference (P = 0.03). Table 5 (see PDF) summarizes the adverse effects or allergic reactions observed in both groups. No significant adverse reactions were observed in either treatment group, although mild irritation was reported by a few participants in both groups. Both treatments had similar and minimal adverse effects and no significant differences were found between the groups (P > 0.05). Table 6 (see PDF) presents the results of patient satisfaction and acceptance, based on subjective feedback collected at the end of the study. Satisfaction was rated on a scale of 1 to 5, with 5 being highly satisfied. The Curcumin Group reported higher satisfaction levels, with 18 participants rating the treatment as highly satisfactory, compared to 12 in the Triamcinolone Acetonide Group (P = 0.05).

## Discussion:

This study aimed to compare the efficacy of Curcumin gel and Triamcinolone Acetonide gel in treating minor RAS. Our findings indicated that the Curcumin gel demonstrated superior outcomes in terms of pain reduction, reduction in ulcer size, shorter healing time and higher patient satisfaction. These results are consistent with several previous studies evaluating the effectiveness of Curcumin in RAS treatment. A randomized, placebo-controlled trial by Kilor *et al.* (2025) [19] reported that a 2% Curcumin gel significantly reduces ulcer size and pain intensity compared to a placebo, supporting its therapeutic potential in RAS management. Similarly, Kia *et al.* (2015) found that Curcumin gel was more effective than Triamcinolone Acetonide gel in reducing ulcer size and pain, with fewer adverse effects. These studies corroborate our findings on the efficacy of curcumin in RAS treatment. In contrast, a study by Hasan *et al.* (2024) [20] compared 5% Amlexanox paste and 2% Curcumin oral gel in the management of RAS. While both treatments were effective, Amlexanox paste showed a higher efficacy in reducing ulcer size and recurrence rates. This suggests that while Curcumin is beneficial, other therapies may offer enhanced therapeutic outcomes. Furthermore, a pilot trial by Raman *et al.* (2020) [21] highlighted that both Curcumin and Triamcinolone Acetonide effectively reduce ulcer size, pain and improve patient satisfaction. However, the review also noted that Curcumin's anti-inflammatory properties make it a promising alternative to corticosteroids, which are associated with potential side effects. Additionally, a study by Abbasi *et al.* (2023) [22] found that Salvizan gel, an herbal formulation, was more effective than Triamcinolone Acetonide in pain relief and wound healing in RAS treatment. This underscores the growing interest in herbal alternatives to corticosteroids in managing RAS. Collectively, these studies reinforce the notion that Curcumin gel is an effective treatment for RAS, offering benefits such as reduced pain, smaller ulcer size, faster healing and higher patient satisfaction. However, considerations regarding treatment efficacy, patient preferences and potential side effects should guide therapeutic decisions.

## Conclusion:

Curcumin gel has demonstrated efficacy in treating minor RAS, with outcomes comparable to or exceeding those of Triamcinolone Acetonide gel. Thus, Curcumin gel presents a viable alternative in RAS management. Further studies with larger sample sizes and long-term follow-up are warranted to confirm these findings and establish standardized treatment protocols.
